# Restoring patient trust in healthcare: medical information impact case study in Poland

**DOI:** 10.1186/s12913-021-06879-2

**Published:** 2021-08-24

**Authors:** Roman Lewandowski, Anatoliy G. Goncharuk, Giuseppe T. Cirella

**Affiliations:** 1 Faculty of Management, University of Social Sciences, Lodz, Poland; 2 Voivodeship Rehabilitation Hospital for Children in Ameryka, Ameryka, Poland; 3 Department of Management, International Humanitarian University, Odessa, Ukraine; 4 Faculty of Economics, University of Gdansk, Sopot, Poland

**Keywords:** Influence of information, Physicians, Hospital, Medical profession, Payer, Pandemic

## Abstract

**Background:**

This study empirically evaluates the influence of medical information on patient trust at the physician level, the medical profession, hospitals, and with the payer. Restoring patient trust in a medical setting in Poland appears to be significantly affected due to the COVID-19 pandemic. Patient trust improves results from medical treatment, raises perception of healthcare performance, and smoothens the overall functionality of healthcare systems.

**Methods:**

In order to study trust volatility, patients took part in a three-stage experiment designed via: (1) measured level of trust, (2) randomly dividing participants into two groups—control (i.e., re-examination of level of trust) and experimental (i.e., being exposed to a piece of certain manipulative information), and (3) checking whether observational changes were permanent.

**Results:**

Results indicate that in the experimental group the increase of trust was noticed in the payer (27.7%, *p* < 0.001), hospitals (10.9%, *p* = 0.011), and physicians (decrease of 9.2%, *p* = 0.036).

**Conclusion:**

The study indicated that in Poland medical information is likely to influence patient trust in healthcare while interpersonal and social trust levels may be related to increases of trust in hospitals and in the payer versus decreases in physicians.

**Supplementary Information:**

The online version contains supplementary material available at 10.1186/s12913-021-06879-2.

## Background

In many countries, including Poland, a significant number of COVID-19 infections occur in medical settings. As a result, a lowering in healthcare trust has been widely reported throughout the medical literature [1–5] in terms of other illnesses and diseases not being looked at or treated—especially in a timely manner or at all. Trust is crucial for the smooth functioning of complex systems, particularly in healthcare [6]. It is regarded as an effective tool for evaluating medical performance [7–10] as well as of great importance to a well-functioning healthcare system. This study empirically evaluates the influence of information on trust in physicians, the medical profession, hospitals, and the payer—i.e., the main components of a healthcare system. As such, the motivation of restoring trust in healthcare as a result of the COVID-19 pandemic, or any other circumstance for that matter, is vital to a healthy and viable society. In the direct and concise form, trust could be defined as “a bet about the future contingent actions of others” [11]. In the field of healthcare, trust is commonly understood as “optimistic acceptance of a vulnerable situation in which the truster believes the trustee will care for the truster’s interests” [8], i.e., where a truster is a patient or a citizen and a trustee is a healthcare object.

People’s trust with their physician and, generally, with a provider is vital to the care process. It can modify patient attitude and behavior which can result in better levels of treatment [12–14]. Trust can activate the placebo effect [15], increase acceptance of medical suggestions and compliance with treatment recommendations [16], diminish the risk of underusing medications in response to cost pressure [17–19], and improve motivation to seek help and use preventive care [8, 12]. Moreover, trust enhances communication between doctors and patients [20–22] as well as the perception of efficacy, self-reported health status [23, 24], well-being, and quality of life [5]. Changing attitudes and behaviors associated with patient trust also has a positive impact on healthcare providers. Trust in a provider may reduce the number of conflicts between patients and medical staff [23], diminish the probability of complaining about medical malpractice [25], lower transaction costs (e.g., expenditures that can decrease patient anxiety by using additional diagnostic testing and physician consultation) [23, 26], and increase motivation to recommend the healthcare provider to others [23]. Trust improves the perception of the performance. Studies show that patients with high trust are more likely to perceive performance positively even if it was objectively mediocre [22, 27, 28]. As a result, low institutional trust may cause inefficiency and undermine the legitimacy of health insurance and eventually decrease solidarity [29] as well as overall success of health policy [30]. Thus, an emphasis on the importance of research factors that may affect the level of trust in healthcare become seeming fundamental.

Although there is a rich body of knowledge about factors influencing trust in healthcare, a lack of quantitative research on how specific information is delivered—societally—exists. Understanding how information influences trust could have significant consequences, e.g., visiting a physician, the medical profession in general, hospitals, and the payer. Three points that should be taken into consideration, include: (1) can the level of trust be influenced or even managed regardless of real healthcare performance; (2) what is the ability to revise the data collected, analyzed, and concluded; and (3) are actions taken to increase the trust and criteria of resources (i.e., allocated in healthcare) verified? Trust can be considered both as interpersonal (e.g., trust in a physician) as well as social (e.g., trust in a more abstract sense such as a group of people) [8, 20, 31–33]. Sztompka [11] regards interpersonal and social trust as external boundaries of continuity within which several social categories of trust fall. The boundaries between these categories can be blurred, however, with trust in health systems falling at the far end of institutional trust or system-level trust that overlooks existential security. Hence, the level of patients’ or general public trust is important since “trusting expectation makes a difference to a decision” [34]. As such, scholars emphasize that trust in healthcare is influenced primarily by patient experience, the general public, and mass media [20, 27, 35, 36].

Mechanic [20, 27] claimed that interpersonal trust occurs when there is a possibility of repeated testing over time, i.e., to what extent a person is trustworthy. Interpersonal trust is characterized by intimacy and closeness, and relates directly with people we know personally, whom we recognize by name, and with whom we interact in a face-to-face manner. In healthcare it is represented by the physician-patient relationship. Parsons [37] suggested that a high level of trust in a physician cannot always be explained by the evaluation of objective evidence of trustworthiness since it can also create psychological distress inducive of the illness. This means that in healthcare trust originates from the fundamental psychological attributes of seeking care in a state of anxiety, rather than from provider characteristics or patient personalities [8]. This is consistent with other suggestions that asymmetry of information between a patient and physician [38] in accordance with the logic of professionalism [39] forces patients to trust their doctor. A meta-analysis of 47 studies showed that the correlation between trust and health outcomes is small to moderate [40]. Specifically, trust is moderately correlated with self-rated subjective health outcomes, however there is no correlation between trust and objective, and observer-rated effects [40]. Institutional trust in hospitals, for instance, can indicate consumer appreciation of the organization [29] and affect varying degrees of interpersonal and social trust in its public payers and insurers [13].

Social trust can be influenced by patient experience and the general public’s view of the system [20, 27, 36]. It is trust in abstract objects [41], often referred to as system trust [34] or institutional trust [29], and relates to trust in objects like groups of people, institutions, and health systems. Social trust is more remote, influenced by media exposure, and general reputation [20]. According to Giddens [41], trust in abstract systems provides for the security of day-to-day reliability, but it cannot supply mutuality and intimacy as interpersonal trust such as the physician-patient relationship. Social trust presumes faith in impersonal principles which respond only in a statistical manner when they do not deliver the outcomes which the individual seeks [41]. In particular, social trust may be influenced by patient experience, general public opinion [20, 27, 36], professional institutions and legal as well as regulatory protections [27, 31], institutional guarantees, and government regulation of medical education, protection of patient rights, and healthcare quality supervision [42]. Importantly, these issues need to be conveyed to society as understandable as well as plausibly achievable. This is significant since Maarse and Jeurissen [29] pointed out that low levels of trust are due to the fact that people may not fully comprehend how the healthcare system (e.g., health insurance) works and how money influences physicians and provider behavior. Moreover, they predict that political communication and mass media may play a central role in shaping public opinion, as “facts do matter less than the perception of the facts” [29]. In many countries, a low level of trust is directly correlated with the media, reporting on what goes wrong in healthcare [29] and why. In short, information is one of the critical factors that influences the level of trust in a healthcare system. Maarse and Jeurissen [29] claimed that the central query of “whether better information will indeed translate in higher institutional trust” formulates the foundation of this research. In terms of information and communication theory, a number of studies back the hypothesis that more communication and higher levels of the quality of information received does result in a higher level of trust [43–47]. This paper explores these queries in a Polish context. Poland’s social health insurance system provides access to a broad scope of benefits but there are important coverage gaps, mostly concerning outpatient medicine. The health system tends to rely on hospital care and faces shortages of health employees. Private facilities provide mainly outpatient (i.e., ambulatory) care, whereas the majority of hospitals are public. The National Health Fund (Polish: *Narodowy Fundusz Zdrowia*) (NFZ) is the sole purchaser through its 16 district branches, which manage the purchasing function in their respective districts. The share of gross domestic product that is devoted to health is lower than the European Union average (i.e., 9.8%), with 6.5% allocated to health in 2017 [48]. This discrepancy makes it an excellent candidate to study its institutional trust of the medical profession, hospitals, and payer system as well as interpersonal trust, i.e., over time, at the physician-based level. A breakdown of the study is structured as follows: section 2 frames the research methods, section 3 illustrates the results, and section 4 elucidates a discussion and conclusion on healthcare trust in terms of real performance.

## Methods

### Hypothesis development

In medical trust literature, the most frequently studied components are the physician (i.e., with whom a patient has the most frequent contact), the medical profession, hospitals, payers, and the overall healthcare system [33]. Given this study is based in Poland, objects studied in this research are the same except for the healthcare system. This is important since Polish society often confuses the healthcare system with the payer (i.e., NFZ). In Poland, NFZ operates as a single centralized payer which is the most visible part of its healthcare system. Confusingly, the media and the public often use the words “system” and “payer” interchangeably, thus blurring the difference between these concepts. Hence, a lack of clear separation between these objects makes it impossible to prepare the appropriate information for the intervention and subsequent interpretation of the results. Therefore, to obtain less ambiguous results from this research, only the payer (i.e., NFZ) was selected as it is a better recognized and defined object. Trust level objects, in particular, may vary since trust in different objects may have diverse levels of susceptibility to the supplied information. In consideration of the existing medical literature, the degree to which information influences the trust level of a particular object, may depend on the frequency people encounter it in a particular healthcare system [22] as well as the type of trust being considered (i.e., interpersonal or social). The study considers the following three hypotheses.

#### Hypothesis 1 (H1)

Patient trust in the payer (i.e., NFZ) is most vulnerable to delivered information via the intervention. This hypothesis is based on two foundations. First, patient trust in an insurer (i.e., in this case the payer) is more amenable to change than in a physician [8, 49, 50]. Second, patients in Poland’s healthcare system virtually have no contact with NFZ hence they have no direct experience with it.

#### Hypothesis 2 (H2)

Patient trust in a physician is most resistant to delivered information via the intervention. According to the medical literature, the increase of patient trust in a physician may be associated with the improvement of receiving care promptly [51] as well as perceived physician competence and communication skills [8]. Interpersonal trust occurs when there is a possibility of repeated testing over time—i.e., to determine the extent of a person’s trustworthiness [20]. In addition, there are significant obstacles in delivering information concerning individual physicians, hence, the change of trust in this object, i.e., apart from experience, may originate mostly from the interrelationship between interpersonal and social trust claimed by Parker and Parker [52].

#### Hypothesis 3 (H3)

The fluctuation of patient trust level in the medical profession as well as in hospitals after the delivery of information (i.e., via the intervention) is within the range of trust in a physician and the payer (i.e., NFZ). Patient trust in hospitals and the medical profession are related to patient trust at the physician level [20]. Zheng et al. [23] claimed that patients who trust their physician may worry less about the hospital due to their reliability from their physician to direct them to a suitable place of care, monitor their quality of service, and their clinical outcome. Hall et al. [13] suggested that trust in the medical profession depends to some extent on patients’ previous experiences with their own doctor. Thus, trust in hospitals may be less susceptible than trust in the payer since approximately 15% of Poles have direct (i.e., personal) experiences with hospitals [53] versus 85% with their doctor. Similarly, trust in the medical profession may be more susceptible to delivered information than trust in a physician since the medical profession is more abstract than an individual physician and trust is not based directly on personal experience. However, patient trust in the medical profession should be considered less vulnerable than patient trust in NFZ since trust in the medical profession may be more related to patient trust of a physician—per se [13].

### Study design

The study design, methodological approach, and analysis conform to the 2010 Consolidated Standards of Reporting Trial (CONSORT) checklist [54]. A CONSORT flow diagram for the study is illustrated in Additional file 1. There is significant difficulty in designing a study that assesses the influence of specific information on patient trust level. Understandably, the possibility of controlling information delivery to individuals and measuring the difference of trust level before and after the delivery is challenging. To overcome this problem, a three-stage experiment in an unchanging group of respondents was applied. The experiment was conducted between September 2015 and March 2016 in two medium-sized Polish medical production enterprises within the context of a multi-staged ISO 9000 training program concerning health quality systems. The training was conducted in permanent groupings on all organizational levels, ensuring constant composition of the groups using demographically diverse samples (Table 1). At all stages, the level of trust in a physician, the medical profession, hospitals, and the payer were surveyed. Between the first and second stage, the period of at least 1 month was used to minimize the likelihood that participants could remember their previous responses. In order to determine whether the change was permanent, the third stage was carried out at least 2 months after the second stage.

**Table 1:** Sample description

Demographic factors^a^	Stage 1	Stage 2 Ctrl gr.	Stage 2 Exp. gr.	Stage 3 Part. Ctrl gr.	Stage 3 Part. Exp. gr.
	Participants N (%)				
Sample size	248 (100)	125 (100)	123 (100)	118 (100)	119 (100)
Sex					
Female	122 (49)	61 (49)	61 (50)	58 (49)	59 (50)
Male	125 (51)	64 (51)	62 (50)	59 (50)	58 (49)
No data	1 (0)			1 (1)	2 (1)
Age					
18–30 years	73 (30)	37 (30)	32 (26)	35 (30)	31 (26)
31–45 years	85 (34)	44 (35)	39 (32)	42 (36)	38 (32)
46–60 years	75 (30)	33 (26)	38 (31)	33 (28)	37 (31)
More than 61 years	12 (5)	6 (5)	5 (4)	5 (4)	5 (4)
No data	3 (1)	5 (4)	9 (7)	3 (2)	8 (7)
Income per family member per month (PPP^b^)					
Less than USD 450	57 (23)	22 (18)	29 (24)	24 (20)	31 (26)
USD 451–850	102 (41)	52 (42)	46 (37)	50 (42)	45 (38)
USD 851–1400	50 (20)	29 (23)	20 (16)	27 (23)	21 (18)
More than USD 1401	21 (9)	8 (6)	11 (9)	8 (7)	11 (9)
No data	18 (7)	14 (11)	17 (14)	9 (8)	11 (9)
Health status					
Very well	33 (14)	18 (14)	14 (11)	20 (17)	13 (11)
Well	159 (64)	87 (69)	72 (58)	81 (68)	68 (57)
Average	45 (18)	17 (14)	29 (24)	14 (12)	30 (25)
Bad	3 (1)	1 (1)	2 (2)	1 (1)	2 (2)
No data	8 (3)	2 (2)	6 (5)	2 (2)	6 (5)

^*a*^*Ctrl gr.* control group, *Exp. gr.* experimental group, *Part. Ctrl gr.* participants of the control group in the second stage of experiment, *Part. Exp. gr.* participants of the experimental group in the second stage of experiment, *N* sample size; ^b^purchasing power parity for 2014 adapted from the Organisation for Economic Co-operation and Development [55] data

#### Components of mass media information

In the study, the assumption has been adopted that information delivered by mass media is a contribution to the decision-making process, consisting of two information-based components: (1) statistical-objective and (2) emotional-subjective [56]. Statistical information (i.e., statistical-objectivity) influences the audience when it is comprehensible for the average user, adequate, knowledgeable, trustworthy [57], and presented in a structured manner [58]. On the other hand, the emotional-narrative component of information has a more significant impact on the audience than a statistical one [59, 60]. Moreover, demonstrating statistical data as a graphical representation can increase its impact on decision-making, creating more effective direct stimulus [61, 62].

#### Perception of healthcare in Poland

Surveys conducted throughout Poland indicate a wide discrepancy between the general public trust in healthcare and individuals using healthcare frequently [53, 63]. Responsibility for a lower level of trust from non-users may arise due to a highly correlative link from negative information concerning healthcare disseminated by the media [53, 63], i.e., as a side-effect of the system’s rapid change and political competitiveness [64, 65]. Poland’s healthcare is under constant reform, trying to adjust the post-communistic system to Westernized standards, which leads to conflicts between interest groups struggling to protect their current interests and efforts to try and obtain better access to public funds. The primary result of this strife is negative media output.

#### Design of the information package used

The information package presented to participants was designed in a manipulative manner by presenting Poland’s healthcare as superior (i.e., in a better light) compared to other countries. All the provided data were drawn from the World Health Organization and Eurostat in which particular indicators were chosen in such a way that Poland was a top healthcare provider. The information package is not in-line with the main “climate” currently being portrayed by Poland’s mass media. The contrast was designed by comparing the United States as the country with the highest spending rate on healthcare in the world and other wealthy Western European countries as well as with some former communist countries which were on the same economic level before their collapse in 1989. The information package consisted of two types of information. First, it targeted an emotional-narrative by starting off with the first 30 min of the film “Sicko” directed by Michael Moore, dubbed in Polish [66] (Additional file 2). Second, statistical-objectives were stressed to elucidate data from official international health statistics presenting a number of graphs mostly illustrating country-related expenditure and data concerning medical errors in American hospitals. The information package was prepared in a contradictory manner to the mainstream point of view.

The impact of the film tested whether it would impact patient trust for the payer since it focuses on healthcare insurance and system-specific aspects. It might also affect the medical profession, especially when it portrays a medical doctor making a public confession that she had one primary duty—to use her medical expertise for the financial benefit of the insurer, stating “… doctors at health insurance companies actually are responsible for the death of patients” [66]. The data regarding medical errors in the United States informed participants about the inevitability of medical risk. As a result, this was supposed to increase the positivity and perception of the performance of Polish hospitals as well as show other countries as less forward-thinking. Moreover, indicators such as standardized death rates for specific cancers or ischemic heart disease illustrated Poland as a comparable alternative to countries spending several times more on healthcare, hypothetically influencing all three objects excluding individual physicians since provided information could not be directly linked to each of the participant’s personal doctor. To strengthen the impact of the statistical information all of the indicators were displayed in three illustrative charts, i.e., function of per capita total expenditure on health, total expenditure on health as a percentage of gross domestic product, and general government expenditure on health as a percentage of total expenditure on health. Next, questionniares concerning the seriousness of medical error in a local hospital and seriousness of medical error from medicine prescribed by a doctor in the European Union was exhibited (Additional file 3). During the intervention all of the indicators were discussed relative to the object—i.e., for a physician, the medical profession, hospitals, and the payer and their influence and level of expenditure on healthcare performance was discussed. Influence of other factors like lifestyle, environment, and human biology were also debated.

### Dependent variables and experimentation

Given the lack of scale, the study’s questionnaire adopted a five-point subscale developed and tested by Ozawa and Sripad [67] and Dugan et al. [68]. To estimate the level of trust in the payer and hospitals, four- and three-point scales developed by Egede and Ellis [69] were applied. The scales were derived from (1) Dugan et al. [68]. (i.e., trust in a physician is the “patient trust in a physician” subscale and trust in the medical profession is the “patient trust in the medical profession” subscale) and (2) Egede and Ellis [69] (i.e., trust in NFZ is the “trust in health care payers” subscale and trust in hospitals is the “trust in health care institutions” subscale). The questionnaire was translated from English to Polish. To ensure authenticity and accuracy of the translations, they were translated back to English by a secondary translator to check whether the meanings remained the same. For each question, a Likert scale was used, and respondents were asked to choose an answer from the following range: 1—Strongly disagree, 2—Disagree, 3—Neutral, 4—Agree, and 5—Strongly agree. The adapted questions from Dugan et al. [68] and Egede and Ellis [69] used for this study can be found as Table 2.

**Table 2:** Questionnaire, adapted from Dugan et al. [68] and Egede and Ellis [69]

Indicator	Strongly disagree	Disagree	Neutral	Agree	Strongly agree
Patient trust in a physician^b^					
^a^Sometimes Dr. __[*insert name of doctor*]__ cares more about what is convenient for him/her than about your medical needs.					
Dr. __[*insert name of doctor*]__ is extremely thorough and careful.					
You completely trust Dr. __[*insert name of doctor*]__‘s decisions about which medical treatments are best for you.					
Dr. __[*insert name of doctor*]__is totally honest in informing you about all of the different treatment options available for your condition.					
All in all, you have complete trust in Dr. __[*insert name of doctor*]__.					
Patient trust in the medical profession^b^					
^a^Sometimes doctors care more about what is convenient for them than about their patients’ medical needs.					
Doctors are extremely thorough and careful.					
You completely trust doctors’ decisions about which medical treatments are best.					
A doctor would never mislead you about anything.					
All in all, you trust doctors completely.					
Trust in health care payers^c, d^					
Health care payers are good at what they do.					
When needed healthcare payers will pay for you to see any specialist.					
When questioned about what treatments are covered healthcare payers are honest with their answers.					
Healthcare payers will pay for everything they are supposed to, including treatment that is expensive.					
Trust in hospitals^c^					
^a^Hospitals only care about keeping medical costs down and not what is needed for my health.					
Hospitals provide the highest quality in medical care.					
When treating my medical problems, hospitals put my medical needs above all other considerations, including costs.					

^a^negatively worded item is reverse coded; ^b^Dugan et al. [68]; ^c^Egede and Ellis [69]; ^d^the Polish translation of the word “payers” was changed into the singular form “*płatnik*”, i.e., since there is only one payer in the Polish healthcare system

In the first stage of the experiment, for the entirety of the participants, the level of trust in a physician, the medical profession, hospitals, and the payer were measured. In the second stage, participants were randomly divided into experimental and control groups of equal size and characteristics. In the control group, re-examination of the level of trust was surveyed, while in the experimental one (i.e., before the questionnaire) participants were shown the information package. In the third stage, the questionnaire was performed again to observe if any change in trust was observable. The manipulation-based check in this experiment is based on observation as well as whether participants believed the intervention. It was performed by a number of research assistants, who monitored participants and occasionally intervened when they were distracted. The research assistants also asked questions to monitor participant attention and continually verified if all participants, in a similar manner, had understood the information.

Statistical analysis was performed using Statistica Version 13 software with the *p*-value of 0.05 (i.e., a 95% level of significance). Considering the experiment consisted of three stages in which two of them were performed in two groups, in total, a comparative examination of five tests had to be carried out. To validate the significance of differences in the mean value of the various stages the Tukey post hoc test was applied. This test provides sounder, more conservative results than the comparison of pairs via the use of analysis of variance.

## Results

**Fig. 1:**
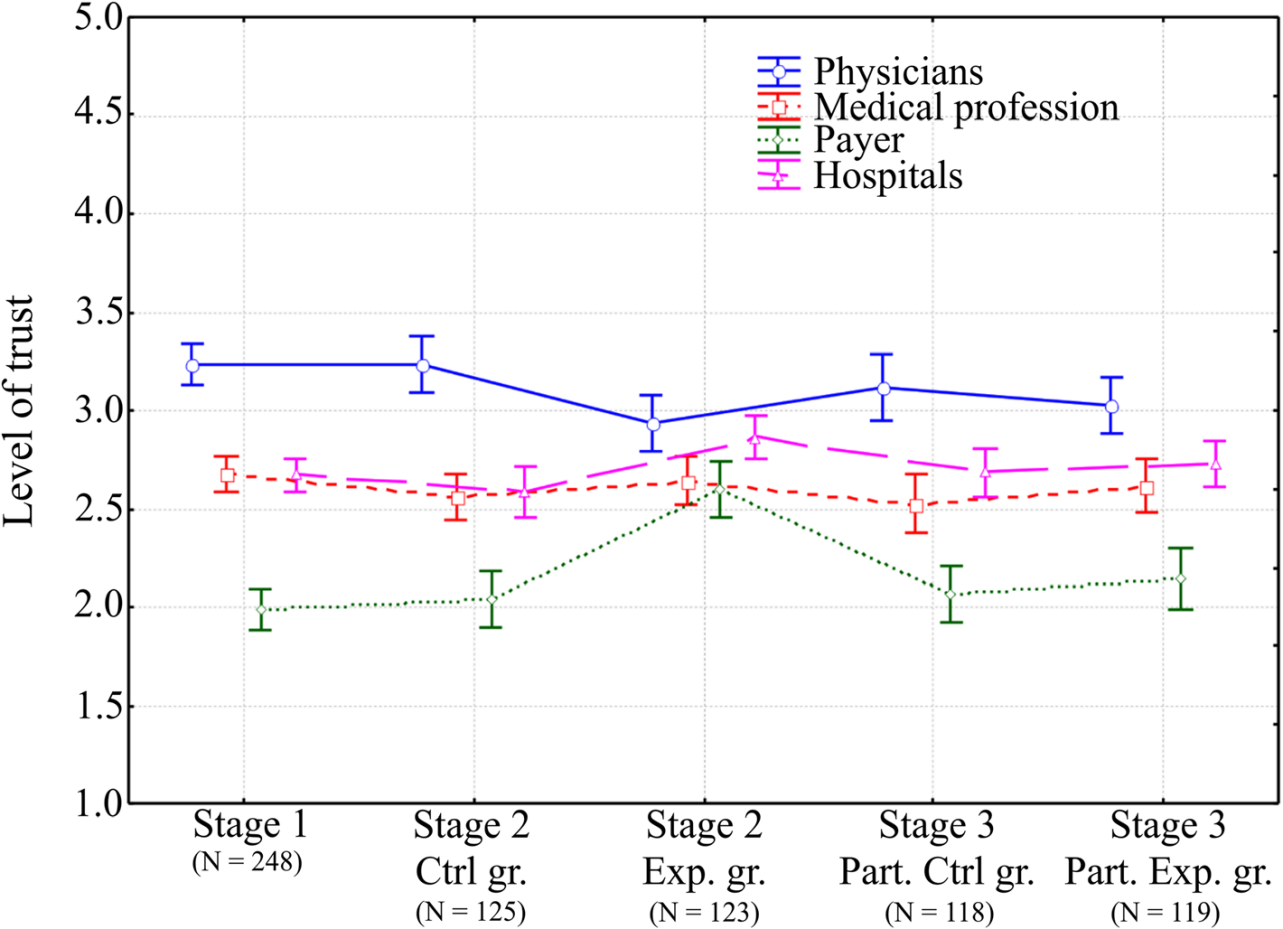
Mean value of the level of trust and confidence interval (i.e., 95%), adapted from . Ctrl gr. = control group; Exp. gr. = experimental group; Part. Ctrl gr. = participants of the control group in the second stage of experiment; Part. Exp. gr. = participants of the experimental group in the second stage of experiment; N = sample size

Lack of statistically significant differences in the level of trust between the first stage of the study and the control group in the second stage showed that between the two questionnaires no factors had influenced the initial level of trust. As a result, changes observed in the experiment were consequential to the information delivered during the study. The results obtained from the Tukey post hoc test indicated substantial increases of trust in the experimental group in the second stage (Table 3). Comparatively, the control group can be observed first by the payer (i.e., an increase of 27.7%, *p* < 0.001) followed by hospitals (i.e., an increase of 10.9%, *p* = 0.011), and, surprisingly, also by physicians (i.e., a decrease of 9.2%, *p* = 0.036). It is worth noting that trust in the medical profession and hospitals presented a very similar result (i.e., *p* > 0.05) during the entirety of the experiment, except for the experimental group after the delivery of the information package where trust in hospitals increased (Fig. 1). Moreover, in the experimental group, trust in the payer reached a similar level to the medical profession (i.e., *p* > 0.05) but trust in physicians decreased and converged with the increased level of trust in hospitals.

**Table 3:** Results of Tukey post hoc analysis

Healthcare type^a^	Stage of the experiment			
	Stage 2, Ctrl gr. (*N* = 125)	Stage 2, Exp. gr. (*N* = 123)	Stage 3, Part. Ctrl gr. (*N* = 118)	Stage 3, Part. Exp. gr. (*N* = 119)
	*p*-value			
Physicians				
Stage 1 (*N* = 248)	1.000	0.032	0.808	0.277
Stage 2, Ctrl gr. (*N* = 125)		0.036	0.829	0.298
Stage 2, Exp. gr. (*N* = 123)			0.416	0.911
Stage 3, Part. Ctrl gr. (*N* = 118)				0.907
Medical profession				
Stage 1 (*N* = 248)	0.735	0.998	0.502	0.975
Stage 2, Ctrl gr. (*N* = 125)		0.896	0.995	0.975
Stage 2, Exp. gr. (*N* = 123)			0.701	0.998
Stage 3, Part. Ctrl gr. (*N* = 118)				0.857
Payer				
Stage 1 (*N* = 248)	0.993	0.000	0.957	0.595
Stage 2, Ctrl gr. (*N* = 125)		0.000	0.999	0.840
Stage 2, Exp. gr. (*N* = 123)			0.000	0.000
Stage 3, Part. Ctrl gr. (*N* = 118)				0.946
Hospitals				
Stage 1 (*N* = 248)	0.851	0.165	1.000	0.976
Stage 2, Ctrl gr. (*N* = 125)		0.011	0.774	0.512
Stage 2, Exp. gr. (*N* = 123)			0.258	0.489
Stage 3, Part. Ctrl gr. (*N* = 118)				0.994

^*a*^*Ctrl gr.* control group, *Exp. gr.* experimental group, *Part. Ctrl gr.* participants of the control group in the second stage of the experiment, *Part. Exp. gr.* participants of the experimental group in the second stage of the experiment, *N* sample size

Finally, the statistical analysis demonstrated that in the third stage, the level of trust of all tested objects returned to initial values. However, in none of the studied objects specific to the second stage, the experimental group showed a significantly different level of trust from the participants of the control group in either the second and third stage.

## Discussion

The analyzed experimentation demonstrated that the level of trust in physicians, the payer, and hospitals appeared to be sensitive to submitted information while trust in the medical profession did not. As a result, the research may prove that information, not in-line with the mainstream opinion (i.e., projected from mass media) could have a significant impact. The study, to some extent, also confirmed *H1* and *H3* that trust in the payer is more vulnerable than trust in hospitals. Furthermore, attention should focus on the requirements that decrease the level of trust in a physician, as indicated in Fig. 1. Compared to hospitals, the change is relatively large and in the opposite direction. Taking a closer look at *H2,* this result was not expected since, first, there was no particular information aimed at influencing trust in a physician and, second, the information package was designed to show Poland’s healthcare in a more favorable light. Hence, the present research has attested that social trust in the payer and hospitals may be strongly related to trust in a physician, as claimed by a number of studies [13, 20, 52].

Due to the unexpected results concerning the change of trust in physicians, per se, 1 week after the last stage of the experiment, a meeting was organized with the experimental group to discuss the results. Some participants suggested (i.e., and some agreed with the suggestion) that after they saw the information, they felt less vulnerable and dependent on their physician then before. Earlier accounts had the majority of them emphasizing their physician was working against the deficiencies of the system. After reflecting on the information package after the results were computed they were more convinced that other elements of “the healthcare chain” worked correctly, they felt more secure, and they did not need to trust so much in their physician.

The findings from the study are similar to Hall et al.’s [8] conclusion in which “the greater the sense of vulnerability the higher the potential for trust” and to some extent to Zheng et al.’s [23] suggestion that patients who trust their physician may worry less about the performance of other healthcare components. When people perceive healthcare performance as mediocre, they rely more on their physician (i.e., reciprocal with a higher level of trust). But when they realize that other healthcare components work properly, their sense of vulnerability decreases and, consequently, they lose trust in physicians who supposedly compensate for the deficiencies of other healthcare components. To some extent, this reasoning might be supported by the fact that under the influence of information, i.e., the trust level of a physician meets the trust level of hospitals and approaches the trust level of the medical profession and the payer, there exists a type of hierarchical level of scaling from the physician down. Moreover, this phenomenon may be interpreted differently, in that participants decrease their trust in a physician because they realize their physicians are not bearing exceptional efforts to organize treatment for patients, but operate in an interdependent environment and are equally important as other components of the system. Limitations to this study invites further research to examine the intervention level—i.e., a likeliness it would not affect all objects in the same manner—distorting the results. Still, as the changes in the payer, physicians, and hospitals reported, these objects were assumed susceptible to delivered information. On the contrary, the lack of change in trust in the medical profession may suggest that the object is vulnerable to delivered information but that the intervention may not have been adequate or adequately scaled. Scales used in the study were not tested on the general society Poland-wide, therefore cultural or organizational differences in healthcare between Poland and the United States may have affected the outcome.

## Conclusion

The main conclusions point towards the findings that information can significantly change people’s trust in some components of healthcare regardless of their real performance. This allows for a number of additional inferences to be made [70]. First, trust in healthcare may strongly correlate with the atmosphere in mass media. Second, patient trust in a physician (i.e., at the interpersonal trust level) and social trust in the payer as well as in hospitals may be interrelated also in opposite directions. This means that the increase of trust in hospitals and the payer may correlate with the decrease of trust in a physician. Third, the assessment of healthcare performance [71] based on trust surveys might be misleading, since any change in trust level may not necessarily translate into an immediate modification (i.e., need) of healthcare functionality. Fourth, delivering designed information is likely to influence the perception of healthcare performance. Fifth, the change in trust level may not be durable. Finally, considering previous studies (e.g., Balkrishnan et al. [49] and Van Der Schee et al. [42]), future change in trust is more likely to occur under the influence of information rather than after a genuine change in healthcare performance indicating a long-term conclusiveness even within the bounds of the study’s limitations. In retrospect of the current COVID-19 pandemic, these inferences could be applied to restore trust in healthcare during and after it reaches its end. As such, restoring trust in a medical setting is a contemporary concern that countries alike are and will need to deal with in an ever so changing global health response.

## Supplementary Information

**Additional file 1.** CONSORT flow diagram of healthcare participants. Flow diagram illustrating the enrolment, allocation, follow-up, and analysis of the healthcare participants in the study.

**Additional file 2.** “Sicko” (2007) by Michael Moore. A commentary description of the first 30 min of the film “Sicko” by Michael Moore and motivation for using the film as a part of the intervention package.

**Additional file 3.** Medical questionnaire responses from the European Union. Answers to the two questions: (1) “Have you or a family member suffered from a serious medical error from medicine prescribed by a doctor?” and (2) “Have you or a family member suffered from a serious medical error in a local hospital?”

## Data Availability

The datasets collected and analyzed during the study are available in the ResearchGate repository [10.13140/RG.2.2.23077.12004].

## Abbreviation

NFZ: Polish Health Fund (Polish: Narodowy Fundusz Zdrowia)
