# Supplementary figures and images for: Restoring patient trust in healthcare: medical information impact case study in Poland
Source: BMC Health Serv Res. 2021 Aug 24;21:865. doi: 10.1186/s12913-021-06879-2 (PMC8383260; doi:10.1186/s12913-021-06879-2)

Additional file 1—CONSORT flow diagram of healthcare participants

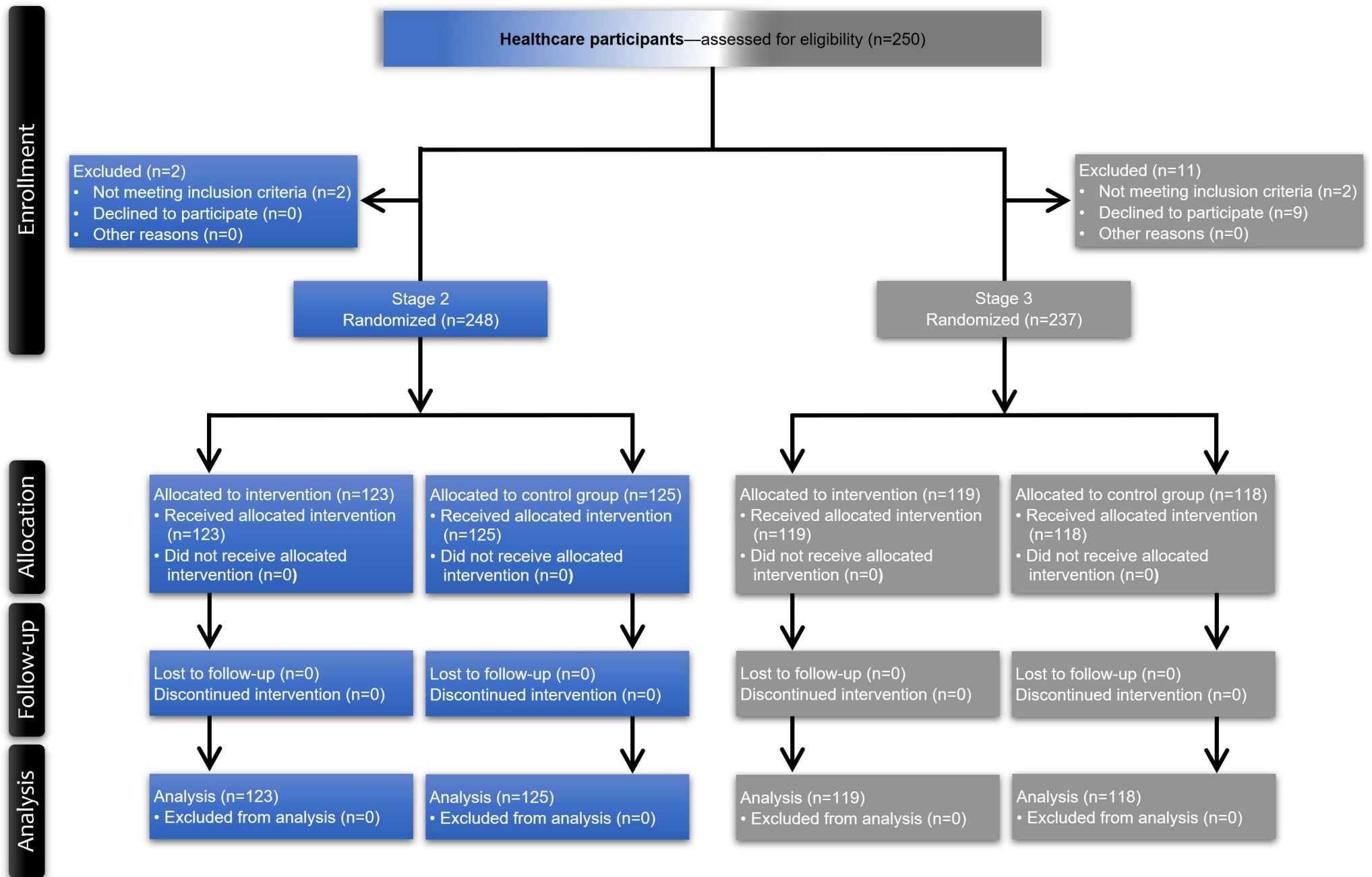

Supplement: Supplementary file 1 — Additional file 1. CONSORT flow diagram of healthcare participants. Flow diagram illustrating the enrolment, allocation, follow-up, and analysis of the healthcare participants in the study. [file 12913_2021_6879_MOESM1_ESM.pdf]
