# Supplementary material for: Restoring patient trust in healthcare: medical information impact case study in Poland
Source: BMC Health Serv Res. 2021 Aug 24;21:865. doi: 10.1186/s12913-021-06879-2 (PMC8383260; doi:10.1186/s12913-021-06879-2)
Supplement: Supplementary file 2 — Additional file 2. “Sicko” (2007) by Michael Moore. A commentary description of the first 30 min of the film “Sicko” by Michael Moore and motivation for using the film as a part of the intervention package. [file 12913_2021_6879_MOESM2_ESM.pdf]

**Additional file 2—"Sicko" (2007) by Michael Moore**

**Description of the first 30 minutes of the film "Sicko" by Michael Moore**

The film "Sicko" [1] begins with the presentation of two uninsured people. First, one-person hand stitches their knee wound by themselves, while a second has two fingers cut off their left palm and due to a lack of funds can only afford to have one finger reattached. The narrator comments that there are nearly 50 million uninsured Americans and 18,000 that die every year due to lack of insurance. The narrator announces that the rest of the film is not going to be about the uninsured but about the problems of the 250 million insured Americans. The next thread of the film shows Larry and Dana Smith, a couple over fifty, who have to move from their own home to live in their adult daughter's storage room. Before they fell ill, they had good jobs, but Larry had three heart attacks, and Dana got cancer. Even though they were insured, the co-payment and cost of treatment made them unable to keep their home. A number of sequencing scenes illustrate:

- an old man who is required to work in order to be able to buy pharmaceuticals;
- a young woman who had a car accident and lost consciousness to later find out that her health plan denied her the coverage costs of the ambulance ride to the hospital;
- four women of whom different insurance companies denied covering the cost of cancer treatment; and
- a young girl who was hearing impaired in both ears and her insurance company agreed to paid the costs of a hearing implant for only one of her ears and not the other.

Generally, the analyzed section of the film shows how insurance companies, acting as mainly health maintenance organizations, operate exclusively to maximize profit. Health plan employees also describe the three-stage strategy that their companies utilize; it is as follows: (1) health insurance companies deny insured people who could bring more risk of future expenses, i.e., people who had suffered previously from certain illnesses or people who were far from average, e.g., too thin or with too much body mass, even though they were healthy and young. (2) The companies then deny payment for treatment and (3) if they had to paid, they thoroughly look over the treatment for every possible mistake in terms of medical documentation of the insured person over the previous five years. Such procedures were diligently performed in order to claim back money in case of any oversight, such that, any unmentioned previous illness or symptoms that one may have had and ignored but might have caused the present illness is re-reviewed and revoked.

The film then explores health insurance employees and digs deeply into their work and investigates ethical and moral issues of the companies. A physician, the medical consultant for a healthcare insurer, describes the motivation system employees utilize. She claims that a bonus system exists where bonuses are given out only to medical consultants who have the highest level of payment denials. Another former medical reviewer and medical director at Humana Health Insurance made a public confession that she had one primary duty, i.e., to use medical expertise for the financial benefit of the insurer. At the end of the analyzed section of the film, the narrator states that "doctors at health insurance companies are actually responsible for the death of patients."

**Motivation for using the film "Sicko" as part of the intervention package**

The first 30 minutes of the film "Sicko" [1] was chosen due to common belief that Poles, at least up until a few years ago, still commonly believe that the United States is an exceptional and opportunistic country with excellent standards of living, inclusive of its medical care [2, 3]. Since the late-1800s, Poles have traditionally migrated to the United States [4] and have commonly refer to it as a country that houses (long-lost) relatives and friends from decades past. The tradition of migrating to the United States increasing one's standard of living—in comparison to living in Poland—is still today the norm [5]. The utility of this film is that it—in total—offers a contradictory viewpoint to the norm.

**References**


1. Moore M. Sicko. United States of America: Lionsgate and The Weinstein Company; 2007.
2. Falcettoni E, Nygaard V. A Comparison of Living Standards Across the States of America. SSRN Electron J. 2020. doi:10.2139/ssrn.3539893.
3. Kozaczka GJ. The Story of Polish Immigration: Polish American Studies and Literature. Pol Am Stud.

2020;77:54.
4. Lucille S. The Causes of Polish Immigration to the United States. Pol Am Stud. 1951;8 3/4:85–91. https://www.jstor.org/stable/20147265?seq=1#metadata_info_tab_contents. Accessed 21 Apr 2021.
5. Yardeni E. Americans’ living standards are at an all-time high. Here’s proof. MarketWatch. 2019. https://www.marketwatch.com/story/americans-living-standards-are-at-an-all-time-high-heres-proof-2019-05-02. Accessed 21 Apr 2021.
