# Supplementary material for: Restoring patient trust in healthcare: medical information impact case study in Poland
Source: BMC Health Serv Res. 2021 Aug 24;21:865. doi: 10.1186/s12913-021-06879-2 (PMC8383260; doi:10.1186/s12913-021-06879-2)
Supplement: Supplementary file 3 — Additional file 3. Medical questionnaire responses from the European Union. Answers to the two questions: (1) “Have you or a family member suffered from a serious medical error from medicine prescribed by a doctor?” and (2) “Have you or a family member suffered from a serious medical error in a local hospital?” [file 12913_2021_6879_MOESM3_ESM.pdf]

Additional file 3—Medical questionnaire responses from the European Union

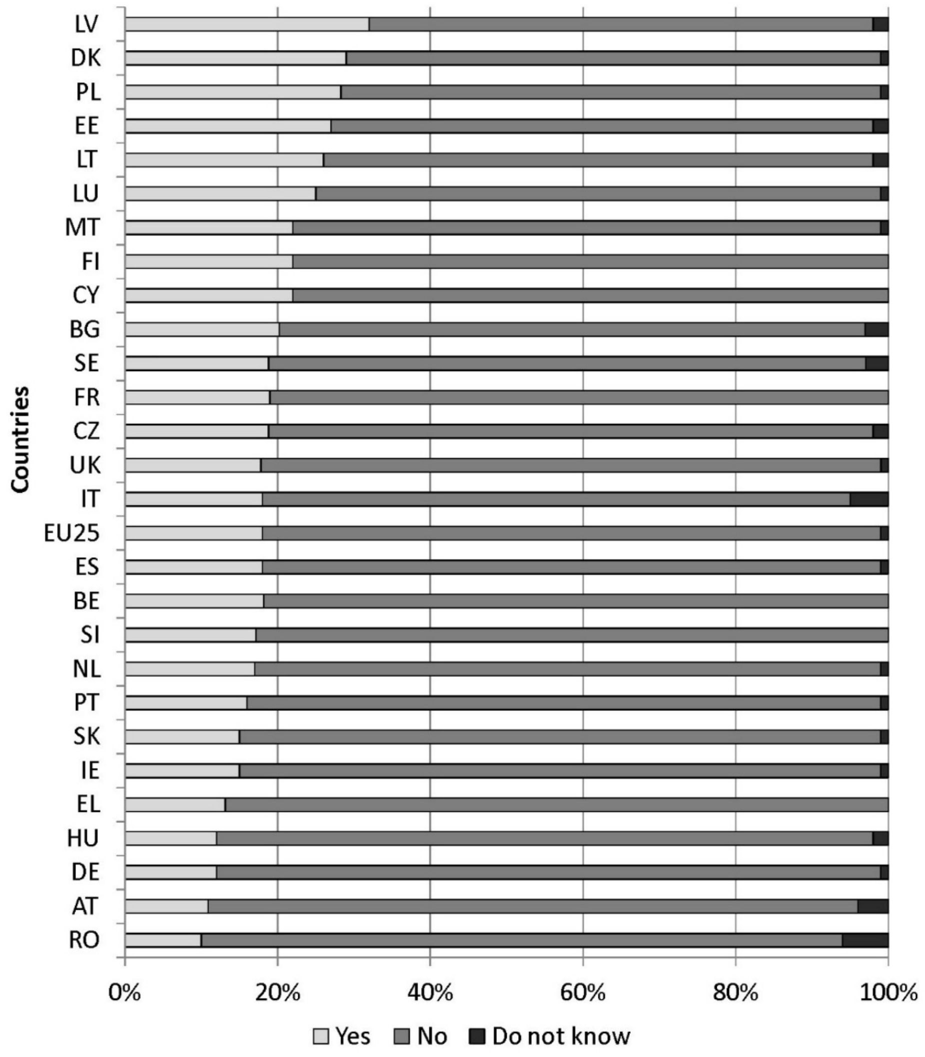

Question 1. Answers to the question: “Have you or a family member suffered from a serious medical error from medicine prescribed by a doctor?”

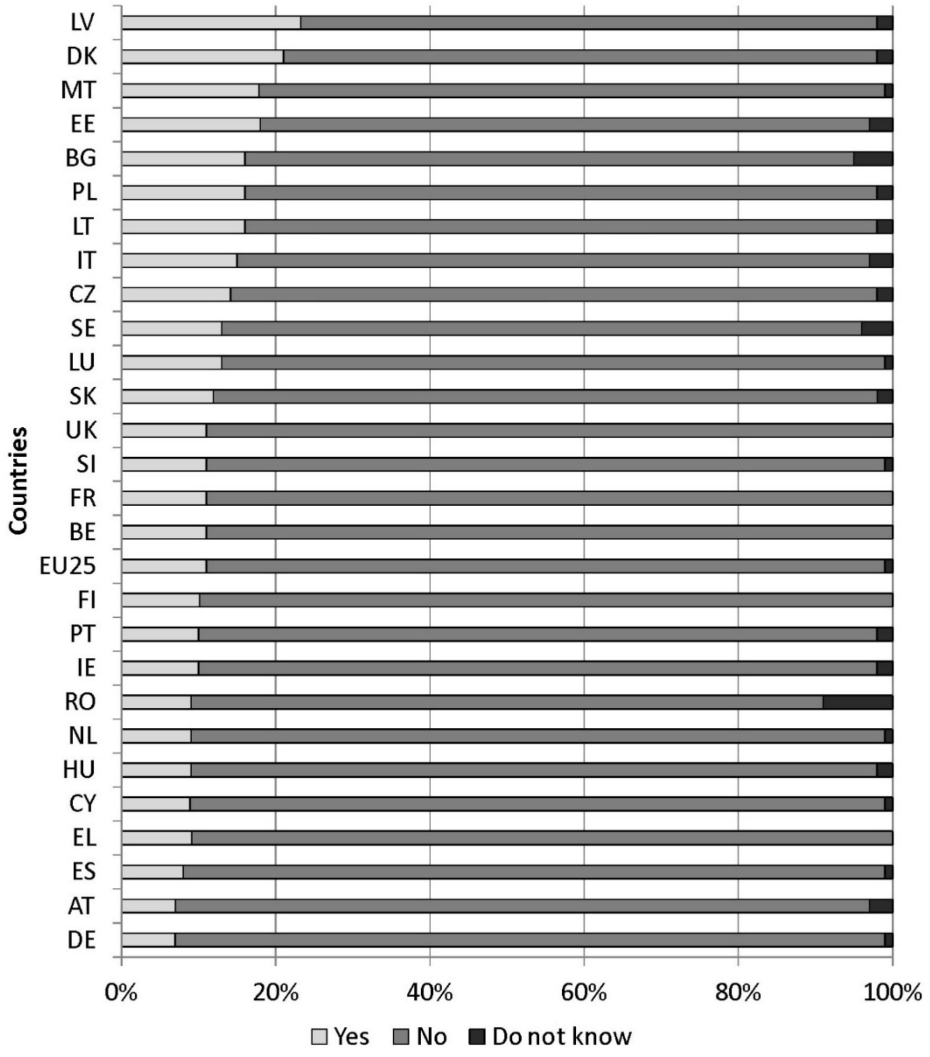

Question 2. Answers to the question: "Have you or a family member suffered from a serious medical error in a local hospital?"
